# The Relationship Between Psychosocial Interventions and Child Wellbeing in Zambia

**DOI:** 10.1007/s12187-022-09982-w

**Published:** 2022-11-02

**Authors:** Vidya Diwakar, Amanda Lenhardt, Emmanuel Tumusiime, Joseph Simbaya, Arthur Moonga

**Affiliations:** 1 Institute of Development Studies, Falmer, UK; 2 ODI, London, UK; 3 World Vision, Federal Way, WA USA; 4 Institute of Economic and Social Research, University of Zambia, Lusaka, Zambia; 5 Frontiers Development and Research Group, Lusaka, Zambia

**Keywords:** Behaviour change, Social resilience, Child wellbeing, Poverty, Zambia

## Abstract

Psychosocial factors contribute to persistence of poverty, but are rarely addressed in poverty reduction programs. We use mixed methods to investigate the relationship between a psychosocial behaviour change approach—empowered worldview (EWV), and investment decisions in children wellbeing among smallholder farmers in Zambia. Three years after exposure to EWV, logistic regression model results suggest that exposure to EWV was associated with an increased probability of parents providing basic needs of children including school fees, clothes, and food. This probability increased with more trainings. Using a matched sample, the average treatment effect on the treated of EWV is positive and statistically significant. Qualitative results reveal EWV enhanced participant agency, spouses’ propensity to work together and with others in the community, and aspirations for themselves and their children. These results point to the prevalence of psychosocial constraints and the need for interventions to sustainably address them to support human development.

## Introduction

There has been a general decline in poverty rates internationally over the last two decades pre-COVID-19, from 27.8% of the global population 2000 to 9.3% by 2017 (WDI, 2021). Even so, a large share of individuals in low- and middle-income countries still live in poverty, with children twice as likely as adults to live in households in extreme poverty (Silwal et al., 2020). Low levels of investment in children, particularly in education, health, and nutrition, in turn have implications on human capital development and intergenerational persistence of poverty. Under-investment in child welfare and long-term wellbeing is often associated with household income poverty (Abraham & Mackie, 2005; Karlan et al., 2013), as well as constraining social norms (Hastings & Schneider, 2020; Munshi & Rosenzweig, 2006), including gender biases against women and girls (Bussolo et al., 2009). There is also growing recognition that child wellbeing might be undermined by psychosocial attitudes—internal behaviours which are the product of the combined impact of social factors and individual behaviours (Wuepper & Lybbert, 2017; Kinsler & Pavan, 2016; Bernard et al., 2014). These behaviours may themselves be affected by experiences of poverty and experience of others in the immediate environment (Bandura, 1977; Beaman et al., 2012).

In response, researchers and development practitioners in low- and middle-income countries are increasingly exploring interventions that can boost aspirations and sense of self-efficacy, to support positive changes in wellbeing. These efforts call for the integration of psychosocial behaviour change approaches in economic empowerment programs. However, such explorations are just emerging in pilot interventions, and their range of application and outcomes are less established (Roelen et al., 2020). One study by Bernard et al. (2014) finds positive effects on psychosocial measures and investments in children’s schooling, which are associated with smallholder farmers watching a documentary on peer successes in agriculture and small businesses. Other studies also point to the positive impact of peer effects on school enrolment and aspirations for children’s education, indirectly for example through access to cable television in India (Jenson and Oster, 2009), and increased interaction with more educated individuals in a study of Mexico’s PROGRESA program (Chiapa et al., 2012).

The objective of our study is to investigate the relationship between a behaviour change intervention and its association with investment decisions in children wellbeing. Our study intervention, the Empowered Worldview (EWV), uses faith-based messages to promote positive mindsets, empowerment, and wellbeing among people in poverty. It was developed by World Vision, a child focused non-governmental international organization. At the time of writing this paper, EWV had been applied in programs in 26 countries in Sub-Saharan Africa, Central America, and southeast Asia, with Christian and other faith communities. In Zambia, the intervention was delivered amongst smallholder farmers as part of an economic empowerment program, the Transforming Household Resilience in Vulnerable Environments (THRIVE). Further details on these interventions are provided in the next section.

We used a convergent mixed methods approach to examine the relationship between exposure to EWV and children wellbeing. Our study analysed cross-sectional, household-level data collected nearly 3 years since the first trainings on EWV intervention among programme participants. Our sample households were selected randomly. The quantitative data analysis plan leveraged the exposure to the EWV intervention to form three study groups: (i) THRIVE with EWV, (ii) THRIVE without EWV, and (iii) no intervention. Moreover, the level of exposure to EWV varied among the THRIVE with EWV group. Though we employed propensity matching methods, we lacked baseline data, and thus some bias due to sample selection might remain in estimates of treatment effects. However, our robust mixed methods approach complements the quantitative data analysis through qualitative insights from life history interviews to uncover in-depth experiences and trajectories of poverty and wellbeing (da Corta et al., 2021).

We frame our assessment within a conceptualisation of “social resilience”, which is typically divided into cognitive (mindset/ behavioural) and structural dimensions, and places emphasis on the role of multi-level social factors in resilience-building (Keck & Sakdapolrak, 2013; Paton et al., 2013; Cutter et al., 2014; Kwok et al., 2016). In its application, social resilience has been used to assess the ability of these factors to help adjust to disasters, environmental variability, and also social change and development more broadly (Keck & Sakdapolrak, 2013). Though related to community resilience, social resilience is distinct; resilient communities do not necessarily exhibit traits of being helpful to others within own and other communities. We discuss in the next section how EWV’s faith-based focus implicitly addresses key cognitive dimensions of “social resilience”, while THRIVE complements this by promoting structural dimensions of social resilience. We argue that this holistic focus on social resilience enables decisions and actions that contribute to improved foundational child welfare and wellbeing within households.

The rest of the paper is organised as follows: Section 2 relates EWV program to key components of a “social resilience” framework, followed by an overview of data in Section 3, and the analytic strategy employed in this paper in Section 4. Section 5 presents the results of the mixed methods analysis, while Section 6 concludes.

## Study Framework

### Study Programme Description

World Vision implemented the EWV approach in Zambia as part of an economic empowerment programming, THRIVE. The THRIVE model is an integrated five-year programme that includes, in addition to EWV, interventions to address external constraints, such as promotion of saving and financial inclusion, business skills development, access to improved agricultural practices, and disaster risk reduction. The THRIVE programme in Zambia was designed to be implemented from October 2016 to September 2021.

The EWV intervention is based on Christian faith messages, which promote a sense of positive attitude and perception regarding identity, hope, visioning, compassion, faith, and social relationships at the individual level and within households and communities. These curricular pillars framed the lessons on:

- Identity: helping people find value in their own identity and realise their creative freedom.
- Visioning: participants are assisted to create a vision for themselves and their families, to set goals with actions that will achieve this vision.
- Compassion: selflessly loving people, which flows from the realization that there will be people who for various reasons, such as age or lack of labour capacity, cannot change their poverty situation by themselves.
- Relationships: focus is on nurturing family and community relationships and promoting understanding of social values such as gender equality and existing harmful social norms.
- Faith: believing and having a strong conviction to achieve given available resource opportunities and developing mindsets to overcome shocks and stressors.

In Zambia, World Vision developed a facilitator’s guide which includes modules on the topics above. The guide includes questions, illustrations, and discussion points for local facilitators and project staff to draw from in meetings with groups and individuals involved in the programme. The modules also include scriptural references and stories to help convey the main points.

Before initiating activities in the project’s target communities, the program team mobilized communities to select local facilitators, also called ‘EWV champions’, in the communities. The project staff and ‘EWV champions’ were then trained in a 3-day workshop on the EWV approach, EWV guide, and facilitation skills. These workshops are considered to be ‘Training-of-Trainers’ as participants are expected to communicate the messages of EWV to other members of their communities, through groups and individually. For example, some of the selected EWV champions were faith leaders, who were expected to share EWV messages through their congregations, and some champions had been leaders of producer groups and savings groups in other development initiatives. The sample frame for this study excluded EWV champions as they were considered highly motivated and likely to provide a more positive outlook of the EWV intervention. Our data show respondents in the sample for our study attended 1–5 training sessions (workshops) on EWV, in the first 6 to 12 months of the THRIVE program. Project staff and EWV champions also followed up with individuals and groups who already participated in the training, but this was not uniform across the project communities and participants.

### EWV and Social Resilience

EWV curricular elements dovetail conceptually with cognitive dimensions of social resilience. Social resilience typically refers to the ability of a social unit or group to collectively cope with or respond to external stresses and disturbances (Kwok et al., 2016). The concept implicitly places a focus on ‘living well together’ in ways that can contribute to improvements in welfare. Its definition is typically disaggregated into cognitive and social dimensions.

Cognitive dimensions reflect people’s attitudes, values, and beliefs, including their sense of community and shared views (Paton & McClure, 2013; Paton et al., 2013). These dimensions map onto EWV curriculum pillars. For example, Paton et al.’s (2013) focus on ‘sense of community’ and ‘social support’ relate to EWV’s emphasis on relationships (within communities as well as families). Their emphasis on psychological preparedness “to develop the capacities to manage stress over time” (Paton et al., 2013) mirrors EWV themes around strengthening participant visions for economic security. A focus on ‘leadership’ skills may relate to aspects of identity and faith to contribute to lasting change for participants, their families, and communities. Through this emphasis on family wellbeing and strengthened relationships within families, EWV has the potential to contribute to increases in household investments in children. Moreover, psychological preparedness can enhance family functioning, and in doing so contribute to child wellbeing through positive parent-child interactions and aspirations for children’s future. Relatedly, forward looking behaviours through improved preparedness have been found to be associated with investments in the future including for children’s education and wellbeing more broadly (Bernard et al., 2014).

**Fig. 1:**
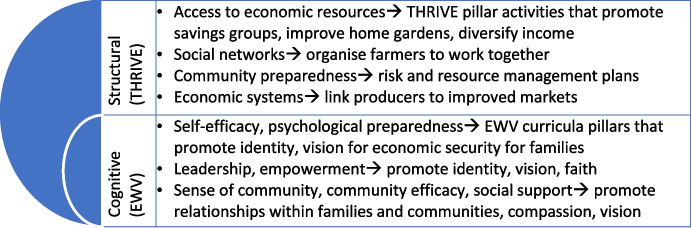
Social and cognitive dimensions of social resilience, in relation to EWV and THRIVE. Source: Author’s illustration based on Kwok et al. (2016) and World Vision’s EWV curricula

Structural dimensions of social resilience include demographic characteristics of people and the communities in which they live, including the actual engagement in social associations or other organisations (Cutter, 2016; Cutter et al., 2014). These dimensions are structural insofar as they require accessing fairly distributed economic resources, and more generally relate to various political, economic, social and cultural factors that affect human agency. Social networks within a community also signify elements of structural social resilience. The economic systems that underlie this relate to THRIVE’s objective to link producers to improved markets, while the community preparedness planning component of structural social resilience relates to THRIVE’s aim to promote risk management plans and natural resource management more generally. THRIVE can thus be conceptualised as strengthening structural dimensions of social resilience. Improved level of connectedness could also intuitively enhance peer effects, which as noted earlier can also enhance investments in children’s education and wellbeing (Jenson and Oster, 2009; Chiapa et al., 2012). Improved access to economic resources and improvements more broadly in welfare are also typically associated with increased spending in health and education, reflecting a relationship more broadly between growth and human development (Stewart et al., 2018).

There are overlaps in these dimensions, where social support (cognitive dimension) may link to social networks (structural dimension), in ways that require the two to be examined together, particularly in its contribution to understanding parents’ investments in child wellbeing. The integration of EWV with the other THRIVE seem to capture the full multi-level potential of social resilience. In particular, it brings questions of power, agency, and participation more centrally into the resilience debate (Keck & Sakdapolrak, 2013). Figure 1 provides examples of these cognitive and structural social resilience indicators. EWV may be conceived as a tool to enhance cognitive, psychosocial elements of social resilience. By strengthening the ability of individuals, families, and communities to have strong relations to live in economic security, EWV may be hypothesized to provide the internal cognitive enablers to contribute to child wellbeing within families and communities. Moreover, when coupled with THRIVE, it can support this mindset with systems support to collaboratively enable economic development of the household unit and carry with it associated improvements in household and individual welfare and promote investments in child wellbeing.

## Data and Research Design

This assessment is based on a mixed methods approach as outlined below. Research was in line with ethical protocols, with approvals provided by the Humanities and Social Sciences Research Ethics Committee at the University of Zambia.

### Quantitative Data

#### Data and Sampling

The dataset is a cross-sectional household random sample collected from THRIVE and comparison communities in Zambia. The study design is quasi-experimental. The survey was conducted from June 29th to July 17th, 2020. It derives from a cluster of four THRIVE Area-wide Programmes (APs) in Mpika AP in Mpika district, Buyantanshi AP in Luwingu district, Mwamba AP in Kasama district, and Katete and Kawaza APs in Katete district. Households targeted for the THRIVE programme in selected APs met an eligibility criterion based on wealth and vulnerability ranking to be extremely poor living on expenditures of USD 1.90 or less a day. Households were asked to respond to a structured questionnaire that includes modules on household demographic attributes, assets, and investments in children’s needs. In addition, there are 10 questions about household characteristics and ownership of assets used to compute the likelihood that the household is living below the poverty line. This probability is derived from the Progress out of Poverty Index (PPI) scorecard, developed by Innovations for Poverty Action.

The sample was constructed to be representative of the intervention eligible individuals across the program area. The evaluation survey relied on a two-stage cluster random sampling approach, with the sample size stratified by Area program.^1^ In the first stage of the two-stage cluster sampling, eight or nine clusters were selected from a THRIVE stratum or area programme using Probability Proportion to the Size method. Villages in the Area Program were considered as clusters, with the size of a cluster being proportional to the number of total project participants in that cluster. In the second stage, 25 households were selected randomly from the list of participant households in each of the selected clusters. This procedure ensured samples in which households had an equal chance of being selected.^2^ This survey was conducted by the University of Zambia’s Institute of Economic and Social Research (INESOR) and overseen by TANGO International.

Comparison households were selected from villages located in the same Wards (civil administrative divisions) as the THRIVE project villages. The comparison villages had been found to be eligible for THRIVE programming in the vulnerability (context) assessment conducted in 2016 to inform geographic targeting of the THRIVE programme. However, not all the villages received direct project interventions because of resource limitations. World Vision provided a list of communities that had been assessed but had not been included in programme interventions. The sampling of comparison households followed the two-stage cluster sample design approach used for the THRIVE participant sample. First, clusters/communities were selected purposefully, with input from THRIVE staff, to ensure communities were far enough from intervention communities to minimize contamination. This was undertaken on considerations that social networks and community leaders can theoretically diffuse social behavior-change messages beyond initial target communities.

In the second stage, households were identified through a random transect walk process (with a sampling interval of at least one household to avoid interviewing neighbours), coupled with screening questions to ensure comparability to THRIVE participants. The screening questions were selected to identify households similar to THRIVE participants. Accordingly, screening questions covered household asset levels three years ago, their main source of income at the time of survey, and participation in programs of NGOs other than World vision. If a household was identified during the sampling interval of the random walk, but after the screening procedure it was determined that it did not fit the targeting criteria, the interview did not proceed. In each selected comparison community, 17–18 households were interviewed, with the total sample size for the comparison group constituting 604 households.

#### Summary Statistics

In total, 1,870 responses were collected. The analysis in this paper relies on the subset of 1,408 respondents who provided responses for at least some of the child welfare questions, indicating that they had children.^3^ Of this sample, 517 were combined THRIVE and EWV recipients (study group 1), 334 respondents were participants in the THRIVE programme who had not received EWV training (study group 2), and 557 are the comparison sample (study group 3).^4^ Table 1 summarises the background characteristics of survey respondents.

**Table 1:** Comparison of sample on some household characteristics

Variables	THRIVE, EWV		THRIVE, No EWV		No THRIVE, No EWV	
	Obs	Proportion	Obs	Proportion	Obs	Proportion
Households able to provide clothes, food, and schooling for children (%)	*495*	0.72	*319*	0.57	*538*	0.46
Households able to provide two sets of clothes (%)	*513*	0.80	*334*	0.72	*555*	0.58
Households able to provide food for children (%)	*501*	0.79	*319*	0.66	*539*	0.54
Households able to provide school fees/ incentives for children (%)	*514*	0.91	*333*	0.81	*552*	0.73
Probability of poverty (%)	*517*	67.92	*334*	74.28	*557*	79.87
Number of training received	*464*	1.64	*333*	0	*552*	0
Household size						
More than 6 members (%)	*517*	0.52	*334*	0.47	*557*	0.51
5 or 6 members (%)	*517*	0.32	*334*	0.34	*557*	0.29
4 or less members (%)	*517*	0.16	*334*	0.19	*557*	0.21
Household head completed primary school or higher	*511*	0.46	*326*	0.32	*513*	0.50
Household head gender	*517*	0.22	*334*	0.30	*530*	0.26
Household primary income source						
Agriculture income (%)	*516*	0.27	*332*	0.37	*550*	0.88
Non-farm income (%)	*516*	0.36	*332*	0.35	*550*	0.09
Micro-business activity income (%)	*516*	0.25	*332*	0.20	*550*	0.01
Remittance, charity, other income (%)	*516*	0.12	*332*	0.08	*550*	0.02
Household assets						
Land (%)	*508*	*0.99*	*329*	*0.95*	*557*	*0.90*
Livestock (%)	*517*	*0.65*	*334*	*0.46*	*557*	*0.38*
Some consumer durable assets (%)	*517*	*0.88*	*334*	*0.73*	*557*	*0.75*

Note: 'Obs' refers to the number of observations (sample respondents) with recorded responses. Source: Authors’ compilation from THRIVE mid-line survey

As the data for the study were collected 2.5-3 years after the EWV intervention, differences observed in Table 1 between groups may be due to the interventions of EWV and THRIVE. For example, we observe large differences in the household’s primary income sources between the ‘no THRIVE, no EWV’ group and the EWV and THRIVE intervention groups. This likely reflect the focus of the THRIVE intervention, which offers business skills development and income diversification, in response to which households may move towards non-farm or micro-business activities as their primary income source.

### Qualitative Data

Qualitative data collection was carried out in three districts (Kasama, Katete and Mpika) covering four APs. The interviews followed a modified life history evaluation methodology (Shepherd et al., 2014), useful in understanding the sequences and processes of wellbeing and factors that contribute to changes in wellbeing over time. Qualitative data collection was carried out between 6 and 23 June 2020. Respondents were identified through stratified purposive sampling. Samples were purposively selected to ensure representation of both sexes and all existing wellbeing (WB) trajectories, including poorest of the poor (WB 1), very poor (WB 2), poor (WB 3), non-poor (WB 4), rich/resilient (WB5), and very rich (WB 6). These WB categories and trajectories were characterized with the community as shown in Table A1 (see Annex). Appropriate COVID-19 precautions were undertaken and written informed consent obtained.

Three qualitative approaches were used, which also help draw out structural and cognitive dimensions of social resilience. Seven key informant interviews on policies and programmes to reduce poverty and sustain escapes were conducted officials from the government (at district level) and community leaders. In addition, three focus group discussions (FGDs) with knowledgeable people (KP-FGD) helped identify key events and processes operating in sampled areas. This was complemented with six FGDs with men and women separately (M-FGD and F-FGD, respectively) from a range of wealth categories. These FGDs tracked respondents’ understanding of poverty mobility, especially since the EWV implementation period-2015–2020. The M-FGD and F-FGD participants also assisted in identification of participants for life history interviews (LHIs). Finally, the 83 LHIs conducted ensured inclusion of participants on different poverty trajectories.

In the qualitative data, the most prevalent WB category at baseline (pre-treatment) among respondents who received the EWV training was WB3 (32 respondents), followed by WB2 (16 respondents), WB4 (13 respondents), WB 5 (3 respondents), and WB1 (1 respondent). This indicates that programme participants in the sample were most likely to be in the poor wellbeing category, with a similar number in either the very poor or non-poor category.

## Analytic Strategy

The independent, mediating, and dependent variables of interest are summarized in Table 2. Exposure to EWV is examined in terms of intensity, proxied by (i) the number of EWV training sessions, and (ii) whether the respondent did not receive any intervention, received only THRIVE, or otherwise received EWV with THRIVE, which helps us to distinguish EWV from THRIVE more broadly. Our outcomes of interest focus on parents/caregivers’ ability to provide for their children (UNICEF, 2007). This indicator is proxied by a series of questions which asks whether, in the year preceding the survey, the respondent was able to provide items for all children (5–17 years) in the household, with or without assistance from extended family, the government, or an NGO. It is a means of measuring whether economic gains at the household level translate into provision for children, for their well-being. These outcomes relate to indicators of child wellbeing used by UNICEF (2007) and other contributions from researchers comparing welfare and child wellbeing in low- and middle-income countries (Arndt et al., 2012).

Finally, through the mixed methods analysis we also focus on certain transmission mechanisms, which closely link to dimensions of cognitive resilience as articulated in Fig. 1. These cover: (1) individual or joint decision making on household expenditures, to reflect cognitive EWV dimensions around participant empowerment as well as their self-efficacy; (2) collaborative economic activities with other community members, to reflect self-efficacy plus social support; and (3) reliance on non-household members during emergencies, to reflect psychological preparedness coupled with a sense of community support. These closely map on to existing literature on cognitive dimensions of resilience as discussed in Section 2.2, such as ‘social support’, ‘psychological preparedness’ and strengthened participant vision (Paton et al., 2013; Paton & McClure, 2013).

**Table 2:** Independent, mediating, and dependent variables of interest used in this analysis

Independent variables	Mediating variables	Dependent variables
• Number of EWV training sessions: 1–2 trainings, 3–4 trainings, 5 or more trainings • Whether the respondent did not receive any intervention, received only THRIVE, or otherwise received EWV with THRIVE	• Female head of household, or husband and wife together, make decisions on minor household expenditures • Respondent engaged in economic activities with members of other communities in week preceding survey • Respondent able to rely on non-household member in long-term emergency	• Care giver was able to provide in the past year all children (5–17 years) in the household, without assistance the follow basic materials needs: 1. two sets of clothes 2. school fees/ incentives 3. food 4. all of the above

To investigate the first relationship relating to the intensity of training, we estimate Eq. (1) as.

1 $${log}_{b}\frac{P}{1-P}={\beta }_{0}+ {\beta }_{1}{X}_{1}+{\beta }_{2}{X}_{2}+\dots +{\beta }_{k}{X}_{k}$$

where P refers to the probability of a child welfare need being provided in the household, ${X}_{1}\dots {X}_{k}$, are covariates, which include household size, education of the household head, gender of the head, household assets, access to loans, the source of household income, area of residence, whether the household participated in THRIVE, and the number of EWV trainings received. Most of these controls are employed to understand how they might influence the type of investments made by households. The key independent variable of interest is the number of EWV trainings received, which is measured as a categorical variable capturing whether the household received: none, 1–2, 3–4, or 5 or more training sessions. Results are reported in Table 3 (Section 5.1).

**Fig. 2:**
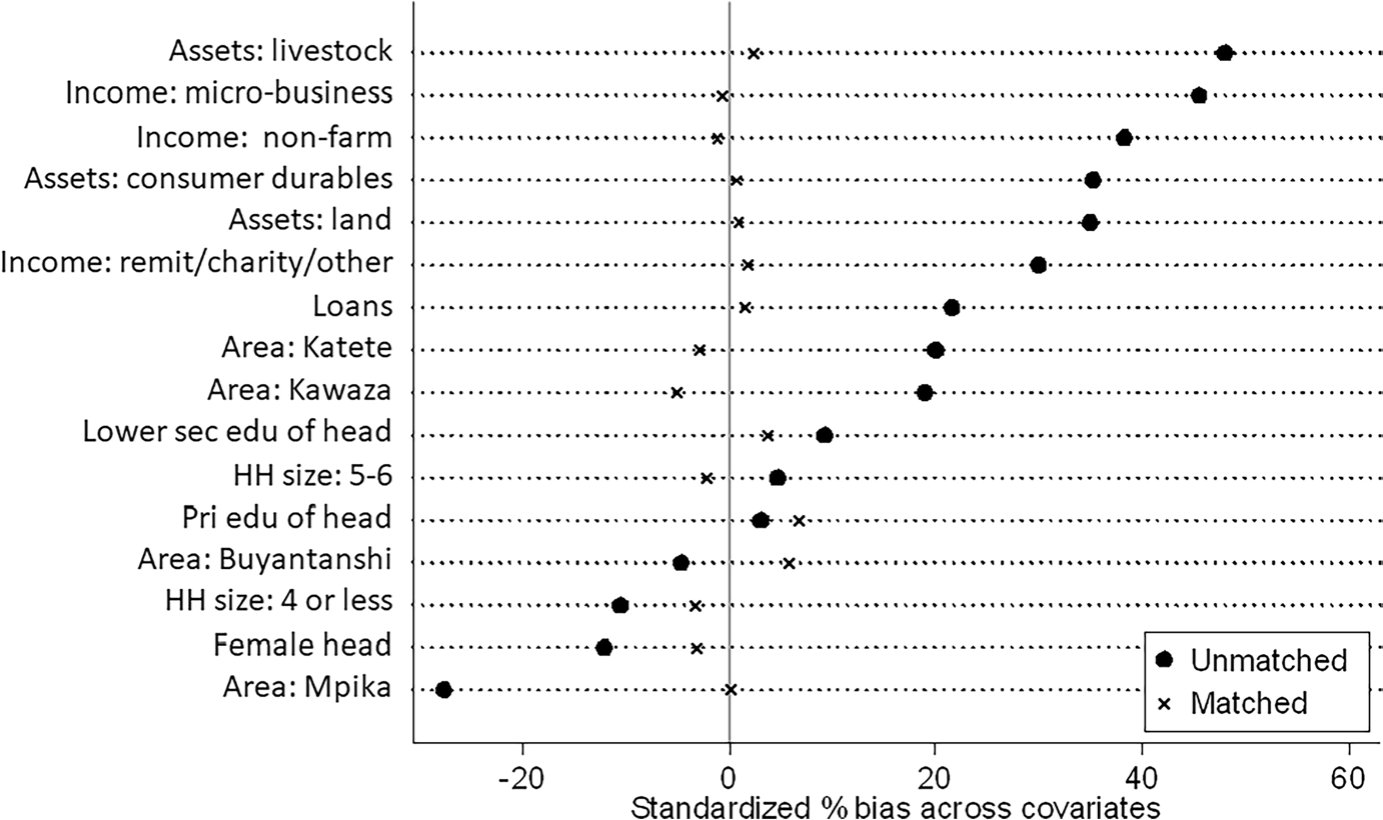
Standardized percent bias across covariates [model outcome = all child needs]

Targeting of THRIVE and EWV interventions was not random, but needs based, and this presents challenges of attribution of observed differences in child welfare outcomes to intervention activities. To help reduce biases due endogeneity, in Section 5.2 we used propensity score matching methods to assess the sensitivity of our results (Rubin, 1997; Rosenbaum & Rubin, 1983). Propensity score matching attempts to provide an estimate of the counterfactual (i.e. the probability of the outcome for the individuals had they not been exposed to the EWV training), by matching on the probability of receiving EWV training.^5^ This propensity score is derived from a logistic regression model relying on the same set of covariates without the THRIVE control. We apply Epanechnikov kernel matching, where all treated units are matched with a weighted average of control units.^6^ We compute 95% confidence intervals using bootstrapping methods with 100 replications. Results of inspection of the quality of matching are summarized in Fig. 2 (Section 5.2).

We then estimated the effect of participation in EWV on child welfare outcomes based on the matched sample. The treatment effect was used to measure the expected effect on the outcome if an individual in the target population was randomly assigned to participate in EWV. We derived the Average Treatment effect on the Treated (ATT) according to Rosenbaum & Rubin (1983):

2 $$ATT=\frac{1}{{n}_{1}}\sum\nolimits_{i\in (T=1)}({Y}_{i1}-\sum\nolimits_{j\in (T=0)}{w\left(i,j\right)Y}_{0j})$$

where Y_1_represents the probability of the outcome Y (provision of child needs) by individuals receiving the EWV ‘treatment’ T = 1, Y_0_ is the counterfactual— the probability of the outcome if the same individuals were not exposed to EWV training, n is the number of EWV participants, j is the individual who did not receive EWV training, and w(i,j) is the weight for an individual who did not receive EWV training, for an individual who did receive this training.

Though this model is useful in understanding how the combined interventions may have affected child wellbeing outcomes, it is certain to pick up some of the impact of THRIVE across both treatment and control groups. To overcome this, we employ a doubly robust estimate that combines regression adjustment with propensity score weighting with the same set of covariates, to examine multiple treatment groups: THRIVE with EWV, THRIVE without EWV, and no THRIVE or EWV. This estimation has the added benefit that only one of the two models need to be correctly specified in order for the estimator to produce unbiased estimates of the mean counterfactual. Finally, in Sections 5.3 and 5.4 we also examine the interaction of these three groups with key behavioural indicators through which EWV is hypothesised to interact with to contribute to the improved ability of household to provide child needs.

The quantitative methods described above are complemented by analysis of the qualitative data. Our aim through this mixed methods, ‘Q-squared’ approach was to get an in-depth understanding of individuals’ experiences in each of the three poverty dynamic groups (Shepherd et al., 2014), as noted in the preceding section. Qualitative data were transcribed and analysed in Nvivo v12. Content analysis was used to identify emerging themes and sub-themes from the data, including in relation to the cognitive and structural dimensions of social resilience articulated in the conceptual framing. Process tracing was then used to observe causal pathways at an individual-case level.

A convergent/concurrent mixed methods approach was used to analyse the combined quantitative and qualitative data. This approach was used in the study design phase, with survey and life history interview instruments developed together to ensure that complementary definitions and measures were applied and that both data sets would support triangulation in the analysis phase. Data analysis was also done in parallel in the first stage of analysis, as presented in Lenhardt et al. (2021). During this process, the quantitative and qualitative research teams regularly reported emerging findings for triangulation and to open further areas of inquiry between data sources. Findings from the quantitative data therefore motivated the exploration of new themes in the qualitative data, which was followed by a final round of quantitative data analysis.

Due to data limitations and the complexity of factors contributing to household and child wellbeing, it is not possible to measure these effects with precision. For example, though we include in our quantitative model factors around assets and income sources, we are unable to control directly for welfare due to data limitations, or for self-selection bias. In addition, we did not have a group who received EWV but not THRIVE, which would have helped improve our understanding of the role of EWV more generally. Finally, there is limited understanding of causality from the quantitative analysis, especially with the absence of panel data. We rely on quasi-experimental methods such as propensity score matching and doubly-robust estimators, alongside process tracing. Even so, our focus is ultimately not on generalizable causal impacts, but instead on understanding relationships between certain levers of program involvement and investments in child wellbeing, while the mixed methods research design also helps contribute to data triangulation on these issues.

## Results: EWV and Investments in Child Wellbeing

### Number of EWV Trainings

Results of the logistic regressions are presented in Table 3. A higher number of EWV training sesssions attended is associated with an increased probability that a household would be able to provide all of two sets of clothes, school needs, and food for children (Table 3, column 1), even after controlling for participation in THRIVE. This is only true though for households that receive at least 3 or more trainings, suggesting that the intensity of engagement with EWV is important. When disaggregating by type of investment, receipt of 3–4 trainings for a household was associated with a higher probability that the household was able to provide food and school fees for their children (compared to no training). Receipt of 5 or more trainings was furthermore associated with a higher probability of the ability of the household to provide clothes and food of their children. The relative importance of EWV thus emerges as a distinguishing factor in contributing to improved child wellbeing in the study locations.^7^

**Table 3:** Average marginal effects (AME) of EWV on child wellbeing outcomes

Outcome: parents reporting providing Variables	1: All (SE)	2: Clothes (SE)	3: School fees (SE)	4: Food (SE)
Number of EWV trainings [ref = no training]				
1–2 trainings	0.0901**	0.0192	0.0650	0.0220
	(0.0356)	(0.0263)	(0.0442)	(0.0350)
3–4 trainings	0.0873***	0.0372	0.0489**	0.0602**
	(0.0245)	(0.0389)	(0.0225)	(0.0265)
5 or more trainings	0.1424	0.1208*	0.0415	0.1128***
	(0.1047)	(0.0689)	(0.0927)	(0.0319)
Participation in THRIVE	0.0434	0.0695	0.0400	0.0168
	(0.0404)	(0.0450)	(0.0254)	(0.0248)
Household size [ref = more than 6]				
5 or 6 members	0.0518*	0.0803***	0.0416	0.0462**
	(0.0314)	(0.0287)	(0.0267)	(0.0201)
4 or less members	0.0537**	0.0912***	0.0182	0.0142
	(0.0240)	(0.0216)	(0.0265)	(0.0205)
Household head education [ref = less than primary]				
Completed primary	0.0287	0.0036	0.0237	0.0197
	(0.0237)	(0.0235)	(0.0287)	(0.0198)
Completed secondary or higher	0.1526***	0.0879**	0.1551***	0.0876***
	(0.0438)	(0.0380)	(0.0382)	(0.0289)
Female headship	-0.1107***	-0.1390***	-0.0818**	-0.0136
	(0.0360)	(0.0291)	(0.0351)	(0.0238)
Household income source [ref = agriculture income]				
Non-farm income	0.0432	0.0475	0.0651	0.0438*
	(0.0420)	(0.0439)	(0.0463)	(0.0235)
Micro-business activity income	0.1357**	0.1389**	0.1392**	0.1255***
	(0.0681)	(0.0579)	(0.0553)	(0.0225)
Remittance, charity, other income	0.1747**	0.1012**	0.1827***	0.1130***
	(0.0711)	(0.0500)	(0.0608)	(0.0287)
Respondent received a loan	-0.0361	-0.0475**	-0.0299	-0.0166
	(0.0225)	(0.0194)	(0.0194)	(0.0232)
Household assets				
Land	0.1596**	0.0459	0.1874***	0.1305**
	(0.0732)	(0.0519)	(0.0664)	(0.0534)
Livestock	0.0433	0.0463	0.0599**	0.0422**
	(0.0349)	(0.0295)	(0.0273)	(0.0177)
Some consumer durable assets	0.2150***	0.1843***	0.2029***	0.1347***
	(0.0251)	(0.0364)	(0.0237)	(0.0327)
Observations	1,212	1,255	1,219	1,253
Psuedo R2	0.1708	0.1695	0.1791	0.2118

Area controls included; Standard errors (SE) clustered at village level; *** *p* < 0.01, ** *p* < 0.05, * *p* < 0.1

Regarding other controls, completion of at least secondary education is associated with a higher probability of providing child needs across the set of outcomes explored. Female headship is assocaited with a lower probability of providing child needs, which may be due to the prevalence of divorced and widowed female heads. There is a positive association between other asset variables and the probability of providing child needs. In particular, livestock and consumer durables are associated with a higher probability of households being able to provide these needs for their children. Loans have a negative relationship with the provision of clothes, though this likely reflects reverse causality whereby loans may be acquired in times of distress. Finally, business activities, off-farm incomes, and other incomes sources generally improve a household’s ability to provide these human capital investments, compared to the reference group of households relying primarily on agricultural income. This likely reflects a context where the majority of people in poverty live in rural areas where farming is the mainstay. It may also partly point to the importance of diversification in the Zambian context, where repeated drought and other stressors may affect the ability of agriculture to contribute to impoved welfare (Shepherd et al., 2021; Diwakar et al., 2021).

### Treatment Effects Through Matched Samples

To test the sensitivity of results, propensity score matching was undertaken. The quality of matching is high, as indicated by visual inspection of common support graphs, and with Rubin’s B and R statistics both falling within conventional values, and the t-test for each covariate having p-values less than 0.05 (Rubin 2001). We present the standardized percent bias across covariates in Fig. 2 for unmatched and matched samples.

Estimates of the ATT are provided in Table 4. Amongst the matched sample, the ATT is positive, suggesting that EWV training was associated with a higher probability that households were able to provide children with school fees/ incentives, food, and clothes in the year preceding the survey. The effect size is largest in terms of enabling households to provide all three items. Within the disaggregated outcomes, the ATT is largest for the provision of school fees. In particular, participants exposed to EWV increased the probability of providing to their children school fees/incentives by 6.4% points, two sets of clothes by 5.1% points, and food by 4.0% points.

**Table 4:** Estimated effects of EWV with THRIVE training on child welfare using propensity score matching, Kernel matching method

Dependent variable:	All	Clothes	School fees	Food
Treatment				
ATT	0.0920***	0.0520*	0.0642**	0.0400**
SE	(0.0320)	(0.0275)	(0.0280)	(0.0189)
Obs	1,254	1,299	1,261	1,297

*p* < 0.01, ** *p* < 0.05, * *p* < 0.1

The results in Table 4 do not match on THRIVE, as doing so necessarily excludes the group of individuals who did not receive THRIVE or EWV training. As such, the ATT is likely to also pick up THRIVE effects. The qualitative data too revealed participants discussing the benefits of EWV partly as a result of THRIVE-based training. Many respondents who reported positive impacts from EWV, also referred positively to learning about improved farming techniques from THRIVE. To distinguish these interventions quantitatively, we adopt doubly-robust IPWRA estimators to measure multi-valued treatment effects.^8^ Results presented in Table 5 show a positive association between EWV training and child welfare outcomes. When compared to the control group who did not receive THRIVE or EWV, moreover, the engagement of respondents in THRIVE without EWV training does not bear a significant treatment effect. The set of results together suggest that EWV with THRIVE provides a human development benefit to households beyond participation in THRIVE alone.

**Table 5:** Estimated effects of EWV training on child welfare using IPWRA

Dependent variable:	Clothes	School fees	Food
Treatment			
ATT (THRIVE with EWV)	0.0973**	0.0675	0.0565**
SE	(0.0483)	(0.0438)	(0.0231)
ATT (THRIVE without EWV)	0.0877*	0.0411	0.0107
SE	(0.0518)	(0.0498)	(0.0309)
Potential outcome means	0.7114***	0.7258***	0.8614***
SE	(0.0461)	(0.0413)	(0.0211)
Obs	1,299	1,261	1,297

*p* < 0.01, ** *p* < 0.05, * *p* < 0.1; we remove the ‘all’ category due to concavity issues in its estimation; control comparison is respondents who did not receive THRIVE or EWV

The next two sections examine the mechanisms through which this relationship may be observed, relying on mixed methods data.

### The Role of EWV in Contributing to Improved Investments in Children

One way in which EWV trained households provided children’s needs was through enhancing behavioural, psychosocial enablers of empowerment. This included factors such as the vision and agency of individuals, especially women. For example, EWV participants were more likely to be confident in their ability to achieve financial goals in life even with their present level of resources, and to think it wise to plan ahead. This vision in turn may have prompted women’s increased contribution to decision-making (Table 6), even amongst households where the official head was recorded as a male member. These aspects point to the importance of agency, power, and participation in decision-making, all components of cognitive social resilience associated with EWV.

**Table 6:** Vision and decision-making by receipt of training

Group	Vision:		Decision-making:			
	Confidence	Wise to plan	Male head	Female head	Joint	Other
THRIVE with EWV	93.58%	52.22%	11.22%	53.19%	30.75%	4.84%
THRIVE without EWV	86.63%	45.59%	17.07%	44.01%	36.23%	2.69%
No THRIVE, no EWV	83.39%	44.46%	16.73%	45.50%	33.63%	4.14%
Pearson chi2(2)	26.5945	7.1357	8.2630	9.0990	2.8221	2.4101
Pr	0.000	0.028	0.016	0.011	0.244	0.300

‘confidence’ refers to the respondent agreeing with the statement that ‘I am confident I will achieve my financial goals in life, even with the available resources’; ‘wise to plan’ refers to the respondent disagreeing with the statement ‘it is not always wise for me to plan too far ahead because many things turn out to be a matter of good or bad fortune’

**Fig. 3:**
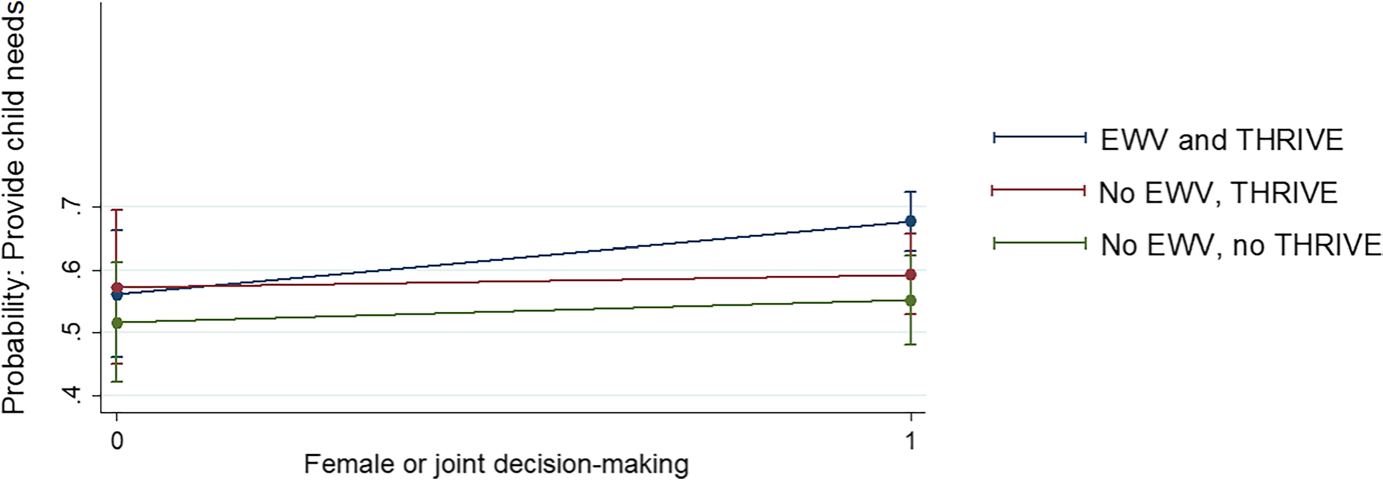
Predicted probabilities of EWV and agency on child wellbeing

We also interacted these behavioural variables with participation in EWV training in a regression model with the same set of controls in Eq. 1. Results point to a link between women’s agency (proxied by female or joint decision-making) for those receiving training, and an improved ability to provide needs of children (Fig. 3), with the difference compared to other forms of decision-making statistically significant for EWV participants.

In the qualitative data, improved agency of women and spousal collaboration was also observed. Jim notes that: “Things have changed in my life and that of my wife, we don’t quarrel but we try to work together and put our ideas that can help us move forward.” In some cases in the qualitative data, this manifested through shared commitment in the value of education. Christy notes:

“I shared my EWV lessons with my family including my children… Right now everyone saves at my home. My children even pay more attention at school because they now have a positive mindset. I was farming before but now do farming as a business and I have added gardening because it is an all year round source of income”*.*

These examples reflects the importance of collaborative spousal and wider family relationships that underpin improved welfare in the household (Shepherd et al., 2021; Diwakar & Shepherd 2021). In this study, this is extended to creating the enabling environment specifically to ensure human development needs of children are met.

**Fig. 4:**
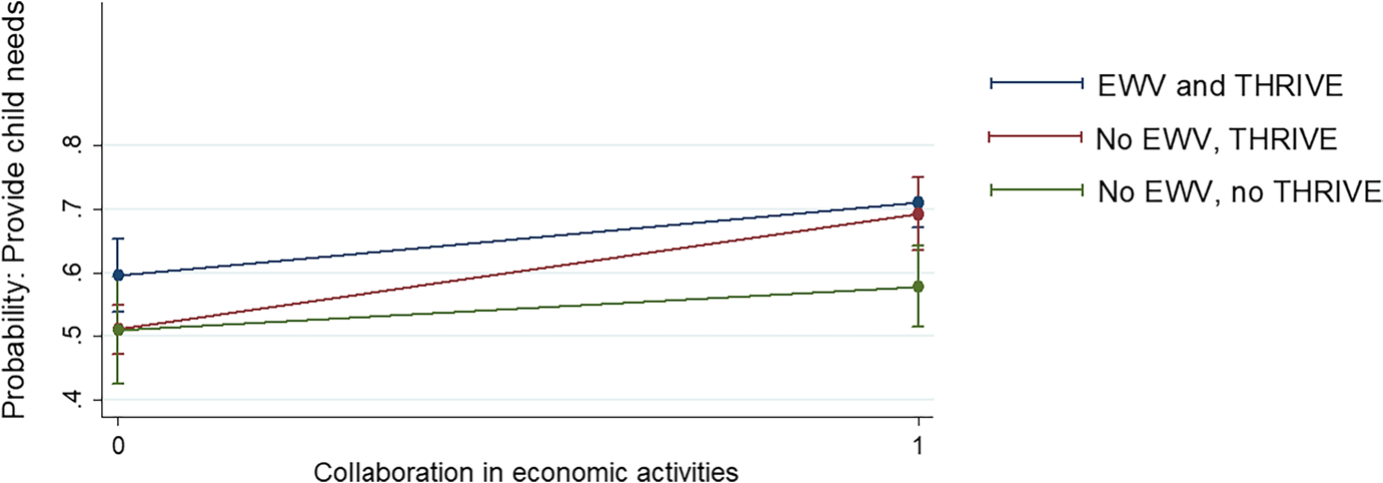
Predicted probabilities of EWV and collaboration on child wellbeing

Collaboration enhanced by EWV training was also observed in relationships beyond the household, where the propensity to participate in group-based economic activities was much stronger amongst EWV respondents than others, in both the quantitative and qualitative analysis. In the quantitative data, participants of EWV training were more likely to engage in economic activities with members of other communities (Table 7), with differences statistically significant. The combination of EWV training with engagement in economic activities with other communities was moreover observed to be associated with a statistically significant increase in the probability of providing needs for children, according to the regression analysis (Fig. 4). Together, these results point to the importance of internal propensities towards participation within social resilience, strengthened through THRIVE’s structural elements, which are observed to contribute to improved child wellbeing.

**Table 7:** Relationships by EWV and THRIVE involvement

Variable	Attend religious service at least 4x in past month	Socialising over food or drinks, in home or public at least 4x in past month	Economic activities with members of other communities in past week	Engaged in self-help groups
THRIVE with EWV	76.22%	52.55%	57.56%	53.0%
THRIVE without EWV	75.45%	50.16%	41.14%	33.3%
No THRIVE, no EWV	75.58%	47.55%	43.53%	18.3%
Pearson chi2(2)	0.0845	2.5984	29.6107	142.2603
Pr	0.959	0.273	0.000	0.000

Similarly, in the qualitative data, before exposure to EWV, Flora’s wellbeing declined in 2016 when her husband fell ill and died. She remembers that it was hard to pay for her three children’s school fees and so they dropped out. Flora was afraid of the future, and her faith went down. Then, in 2018, she undertook EWV training for two days. Flora recalls: “*They said if you cooperate with your friends it’s easy to accomplish your goals. I once went to Mbala where I saw people working together.”* In the same year, she decided to join a savings group and a cooperative, and became a FISP beneficiary. “*We made a group and started to work together, we just choose on our own whom to work with and what you want to do as a group.*” Flora attributes her current wellbeing to the EWV training, a result of which her children resumed their education and she managed to pay for all their school expenses. Engagement in economic activities also extended to collective action around savings (Table 6), where EWV training often resulted in participants being more inclined to join savings groups, generate savings and allocate finances towards improved nutrition and education.

**Fig. 5:**
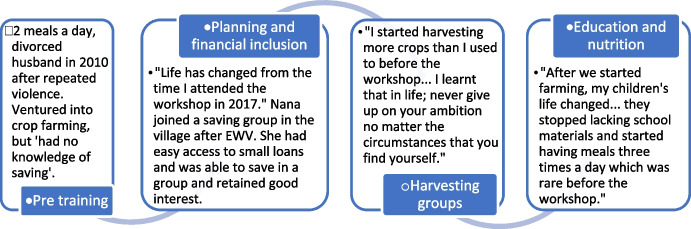
Confidence, training, and improved provision of food and education needs

Financial inclusion often operated alongside skills-based training within THRIVE, that when coupled with EWV’s focus on relationships such as through group-based economic activities helped contribute to improvements in wellbeing sufficient to enhance children’s access to schooling. Nana is one of many who recalls that from EWV, she learned about:

Identity, faith and vision.

How to work in groups and help each other where one fails to do.

How to start up a business and what to put in place for the business to be successful.

How to grow maize; and not to grow it where there is a lot of water because maize does not grow well if water is in excess*.*

Though Nana had ventured into crop farming in 2011, which had slowly helped her family’s welfare improve, she recalls that she had no knowledge of saving and little hope for the future (Fig. 5). Learning around business skills (including savings) in THRIVE, as well as EWV’s group-based focus on livelihood activities including in farming ,was important in helping instil confidence in Nana to “see life differently than before” and enhance agency.

### Underlying Welfare Changes and the Probability of Poverty

Partly as a result of collaboration and better preparedness planning, participants of EWV training generally exhibited a lower probability of poverty compared to other individuals (Table 1). They were also more likely to report increases in income following EWV (Table 8), compared to households receiving only THRIVE without EWV. In the qualitative data, this was sometimes linked to an improved ability to cope with negative shocks that could otherwise prompt impoverishment. Anna notes: “*I did my EWV training in 2018, we learnt that we are supposed to have a vision and get prepared for emergencies. We also learnt that we are supposed to cooperate in a community and share the knowledge, they demonstrated by tying clothes to make one long cloth.”*

**Table 8:** EWV and change in income

Group	Income change in 3 years pre-survey			Ability to rely on non-HH in long-term emergency
	Increase	Same	Decrease	
THRIVE with EWV	50.3	8.68	41.03	80.3
THRIVE without EWV	39.51	9.42	51.06	74.8
No THRIVE, no EWV	N/A	N/A	N/A	73.1
Statistical tests	Pearson chi2(2) = 9.6301; Pr = 0.008			Pearson chi2(2) = 7.6335; Pr = 0.022

**Fig. 6:**
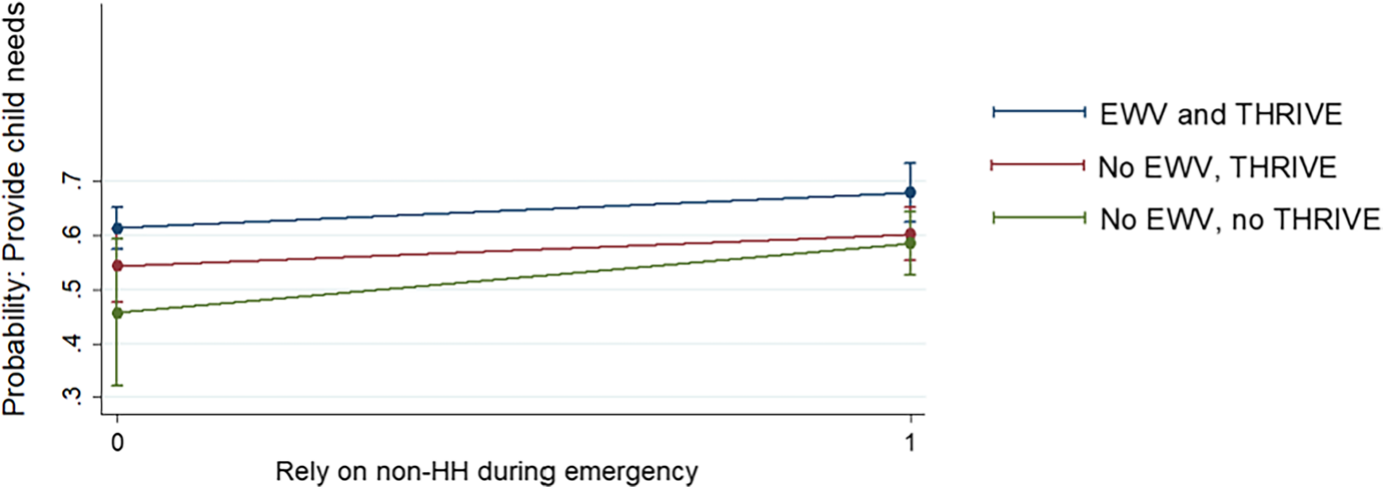
Predicted probabilities of EWV and non-HH reliance on child wellbeing

This preparedness planning was typically observed through increased household savings, but also being able to rely on their EWV-developed networks during emergencies. For example, in the quantitative data, households that received EWV were more able to rely on members beyond their immediate household (such as extended family, or presumably EWV-related social contacts) in a long-term emergency (Table 7). The interaction of this reliance with EWV training was moreover associated with an improved ability of households to provide needs of children (Fig. 6). Together, the mixed methods data suggests that EWV participants were able to internalize perceptions of power and agency in the activities they maneuvered into or out of, and regularly collaborate in economic activities, thus strengthening relationships that might be called on in times of distress. These aspects of social resilience enabled the ability of EWV participants to maintain child wellbeing when faced with negative events.

However, the ability of EWV to help offset declining wellbeing was not universal. In the quantitative data, 41% of EWV households experienced a decrease in income over the three years preceding the survey. Some of this may be attributable to COVID-19 restrictions and associated price rises common during this period (Diwakar, 2020), which affected programme villages. For example, one respondent noted that the pandemic had affected his grocery business, forcing him to increase prices and thus experiencing reduced sales overall amidst depressed demand. Another EWV respondent in the qualitative data who reported a wellbeing decline noted that she could not apply the training without material support. This may point to a critique more generally of resilience programming, related to its oversight of systems’ internal dynamics (Mikulewicz, 2019). According to Pelling (2011, p. 21) the “underlying institutions that generate root and proximate causes of risk, [and] frame capacity to cope” need to be addressed. Indeed, the capacity to cope may be affected by root causes such as different levels of income and health, in turn shaped by social, economic, and political processes and inequalities (Eriksen et al., 2015). Social resilience framings may improve our understanding of social systems and their abilities to respond to change. However, they may still inadequately engage with questions of politics, access to resources, and institutional and system-level inequalities that require transformation (Keck & Sakdapolrak, 2013; Eriksen et al., 2015; Mikulewicz, 2019).

## Conclusion and Implications for Programming and Policy

Our results indicate a positive and significant relationship between a higher number of EWV trainings and the ability of households to provide clothes, education, and food for children. Propensity score matching further confirms the positive relationship between EWV exposure and these higher-level wellbeing outcomes. Keeping in mind the caveats outlined in Section 4, the quantitative models and qualitative insights point to a positive relationship between EWV training and child wellbeing outcomes.

The evidence suggests potential pathways between behavioural changes and localised networks together contributing to improvements in wellbeing. The social and behavioural change aspects within the EWV curricula enhanced participant agency, encouraged households to work together with others primarily in terms of economic engagement, and strengthened their ability to plan livelihoods and contingencies. At the household level, social resilience involved improved collaboration in child and spousal relationships. Beyond the household level, these relational attributes extended to a stronger propensity to engage in savings groups and group-based farming. In these efforts, the identity and vision components of EWV helped individuals exert agency in deciding together how to strengthen livelihoods to help improve human development outcomes of children.

Moreover, the cognitive social resilience components (e.g. confidence, agency, decision-making, and propensity to collaborate) strengthened through opportunities for collaborative activities meant that when emergencies did strike, households were able to more easily draw on pre-established networks to help smooth consumption and needs of children. While this paper focused mostly on improvements in wellbeing, future research would benefit from examining downward processes in more detail when they do occur, to ensure that EWV is effectively tailored to addressing different poverty dynamics to help break the intergenerational transmission of poverty. To this end, multi-sectoral programming that recognizes and responds to the multi-hazard risk profile in study locations is an important step in contributing to sustained improvements in child wellbeing. In this effort, combining the EWV programme with additional complementary interventions in THRIVE is necessary.

Alongside this focus on individual, household, and community-level drivers of wellbeing and the social resilience capacities permeating across these levels, policy and programming would also benefit from stronger links to and within existing institutions and wider systems, while recognising the behavioural aspects that are important in strengthening agency and collaboration. For example, ensuring that there are financial ladders enabling households to move up the finance chain, from savings groups through to cooperatives to bank savings and loans, “with linkages in between, would help the unbanked obtain access to more tools for smoothing consumption and making investments” (Shepherd et al., 2019). Developing system-level resilience through pro-poor infrastructure and policies that address underlying inequalities can help create an enabling environment for programmes to help promote child wellbeing over the long term. Ensuring that these programs are adequately complemented by efforts that support the agency, power, and participation of its participants will help offer a more integrated, sustainable approach to promoting human development.

## Annex: Wellbeing Categories

Wellbeing trajectories were developed through a participatory wealth ranking exercise from gender-disaggregated FGDs. This began with discussing the ‘universal/conceptual based well-being scheme’ (see Table 9) with FGD participants and then understanding their perceptions and further community-specific characteristics of each category.

**Table 9:** Community defined livelihood characteristics by wellbeing category

Wellbeing category	Livelihood characteristics
1: Poorest of the poor	Depend on others for basic needs, eat once a day, live in mud/thatched house, no assets, usually without labour capacity
2: Very poor	Less than three meals and low nutrition, mud/thatched houses, few assets, no resources to fund children’s education, no access to credit
3. Poor	Three meals with higher nutrition, children educated through primary, iron sheet/thatched houses, some assets, some access to credit
4. Non-poor	Eat more than three meals, high nutrition, children educated, cement houses, own assets
5. Rich/resilient	None in the community
6. Very rich	None in the community

## Footnotes

1 The sampling approach and sample size was determined by TANGO International (World Vision’s learning partner leading the general THRIVE project evaluation).

2 In larger clusters the chance that a household will be selected is smaller, but this is offset by the fact that larger clusters have a greater chance of being selected in the Probability Proportion to the Size procedure.

3 31 households that received EWV training in the comparison group were dropped from the analysis.

4 Note that some households responded ‘don’t know’ or refused to answer, and so are missing values not included in this disaggregation.

5 Note that this binary measure also helps overcome measurement errors from the preceding model that may arise in attempting to capture the number of training sessions and their duration.

6 We also examine alternative matching estimators for sensitivity checks. The directionality of results remains the same and are available upon request.

7 We also conduct the regression using ‘receipt of any training’ rather than number of trainings, and separately conduct the regression on the subsample of THRIVE recipients but results are largely similar. We discuss this distinction further in Section 5.2.

8 The IPWRA estimator uses a tolerance of 0.00001 to check the overlap assumption.
